# Characterization of prevalent tyrosine kinase inhibitors and their challenges in glioblastoma treatment

**DOI:** 10.3389/fchem.2023.1325214

**Published:** 2024-01-08

**Authors:** Mahdie Rahban, Sara Joushi, Hamideh Bashiri, Luciano Saso, Vahid Sheibani

**Affiliations:** ^1^ Neuroscience Research Center, Institute of Neuropharmacology, Kerman University of Medical Sciences, Kerman, Iran; ^2^ Physiology Research Center, Institute of Neuropharmacology, Department of Physiology and Pharmacology, Medical School, Kerman University of Medical Sciences, Kerman, Iran; ^3^ Department of Physiology and Pharmacology “Vittorio Erspamer”, Sapienza University, Rome, Italy

**Keywords:** glioblastoma, tyrosine kinase inhibitors (TKIs), genetic heterogeneity, epigenetic heterogeneity, TKIs resistance, blood-brain barrier, computer-aided drug design (CADD)

## Abstract

Glioblastoma multiforme (GBM) is a highly aggressive malignant primary tumor in the central nervous system. Despite extensive efforts in radiotherapy, chemotherapy, and neurosurgery, there remains an inadequate level of improvement in treatment outcomes. The development of large-scale genomic and proteomic analysis suggests that GBMs are characterized by transcriptional heterogeneity, which is responsible for therapy resistance. Hence, knowledge about the genetic and epigenetic heterogeneity of GBM is crucial for developing effective treatments for this aggressive form of brain cancer. Tyrosine kinases (TKs) can act as signal transducers, regulate important cellular processes like differentiation, proliferation, apoptosis and metabolism. Therefore, TK inhibitors (TKIs) have been developed to specifically target these kinases. TKIs are categorized into allosteric and non-allosteric inhibitors. Irreversible inhibitors form covalent bonds, which can lead to longer-lasting effects. However, this can also increase the risk of off-target effects and toxicity. The development of TKIs as therapeutics through computer-aided drug design (CADD) and bioinformatic techniques enhance the potential to improve patients’ survival rates. Therefore, the continued exploration of TKIs as drug targets is expected to lead to even more effective and specific therapeutics in the future.

## 1 Introduction

GBM, defined by histopathologic necrosis and endothelial proliferation features, is an aggressive primary brain tumor with a median survival of fewer than 15 months despite surgical resection, radiation, and chemotherapy, in adults (Thang et al., 2023; Wälchli et al., 2023). There are no known risk factors for GBM, and it occurs without warning signs. Furthermore, its incidence increases with age, with white ethnicity being more commonly affected than black ethnicity, and males being more affected than females (Newton et al., 2018; Grochans et al., 2022; Dain and Zhu, 2023). Considering the invasive nature of GBM and resistance to therapies, recurrence is observed after treatments (Sareen et al., 2022). Large-scale genomics and proteomics analysis demonstrated the proteins and pathways associated with the resistance mechanisms responsible for the recurrence of GBM (Shergalis et al., 2018). One promising avenue for cancer treatment involves the use of tyrosine kinase inhibitors (TKIs) (Bagheri et al., 2022; Li et al., 2023; Zhang et al., 2023).

Genome-wide studies have revealed that cancer initiation, promotion, progression, as well as recurrence are casually associated with kinase mutations (Bhullar et al., 2018). Kinases are enzymes that catalyze the transfer of a γ-phosphate group from ATP to the hydroxyl group of tyrosine, serine, or threonine residues. Around 538 kinases are encoded in the human genome and can activate protein functions to maintain cellular function (Nayak et al., 2022). According to the Cancer Gene Census (CGC), protein kinases are the most prevalent protein family encoded by cancer genes, with 27 out of 291 cancer genes encoding protein kinases (Futreal et al., 2004). The complete set of protein kinases (kinome) has emerged as an appealing target for therapeutic strategies for human malignancies (Fleuren et al., 2016). Several studies reported that the tumor progression and therapy resistance are subsequently related to overexpression and mutation of TKs that activate many critical downstream pathways in GBM (Bolcaen et al., 2021; Peller et al., 2023). In GBM, certain well-characterized mutated TKs are the epidermal growth factor receptor (EGFR), vascular endothelial growth factor receptor (VEGFR), and platelet-derived growth factor receptor-α (PDGFR-α) (Fleuren et al., 2016; Brar et al., 2022).

Following G-protein-coupled receptors, kinases are the second most targeted proteins for various types of cancer treatment (Jackson et al., 2019). Tyrosine kinase inhibitors (TKIs) are small molecules that selectively inhibit the activity of specific TKs, which are enzymes that play a central role in cell signaling pathways. In GBM, the most common TKs targeted by TKIs are EGFR and VEGFR (Li et al., 2023; Long et al., 2023; Smolenschi et al., 2023). Different TKIs were targeted in the cancer phases I, II, II, III, and IV in clinical trials for 20 years (Huang et al., 2020). Studies are currently underway to identify and validate drug targets; however, many of these targets have failed to demonstrate efficacy in clinical trials, mainly due to several challenges. These challenges include issues such as limited permeability through the blood-brain barrier (BBB), the inherent heterogeneity of GBM, immunosuppressive tumor microenvironment, and the development of resistance to TKIs (Aldaz and Arozarena, 2021; Majd et al., 2021).

GBM is a highly heterogeneous cancer, and different subtypes may have different signaling pathways and molecular profiles. This makes it difficult to identify the most appropriate TKIs for each patient (Olar and Aldape, 2014). Many TKIs have poor BBB penetration, making it difficult to reach therapeutic concentrations in the brain (Bhowmik et al., 2015). GBM cells can develop resistance to TKIs through various mechanisms, including mutations in the targeted TK or activation of alternative signaling pathways (Tilak et al., 2021).

The present work discusses the types of gliomas and the molecular mechanism of TKIs, the physicochemical properties of TKIs required to pass through the BBB, and the characterization of TKI-targeted drugs that have been reported in GBM clinical trials. Finally, the potential of the new generation of TKIs as promising therapeutics will be discussed, including their effectiveness and potential for minimizing off-target effects and toxicity.

## 2 Gliomas subclassification

Gliomas are classified into four grades according to their aggressiveness and malignancy by WHO (Ratti et al., 2022). The tumors with low proliferative potential are classified into grade I while Grade II gliomas are characterized by infiltrative capacity and low proliferative activity. These tumors tend to progress to grade III, which is known as anaplastic glioma andshows histological evidence of malignancy. Finally, glioblastomas with signs of necrosis and microvascular proliferation are classified in grade IV as the deadly glioma with a median survival of 12–15 months after diagnosis (Molinaro et al., 2019; Delgado-M et al., 2020).

Verhaak et al. (2010) classified GBMs based on multi-dimensional genomic data into four subtypes of abnormalities in PDGFR-α, EGFR, isocitrate dehydrogenase 1 (IDH1), and neurofibromatosis type 1 (NF1) (Verhaak et al., 2010). These subtypes contain proneural, neural, classical, and mesenchymal classes. The enrichment in the oligodendrocytic shows proneural. The association with oligodendrocytic and astrocytic display neural. The murine astrocytic signature is associated with the classical group. The mesenchymal phenotype, Schwann cell markers, and microglial markers exhibit mesenchymal (Verhaak et al., 2010; Jackson et al., 2019). However, the classification of GBMs remains controversial owing to the heterogeneity of tumors.

Traditionally, glioblastoma classification had been based on histological features, though this approach frequently lacked precision. In 2016, the WHO revised glioma classification utilizing molecular parameters to define tumor identities. The most frequent and invasive type of glioma is glioblastoma which is divided to three groups based on the status of the IDH gene. The primary or *de novo* group of glioblastoma contains wild-type IDH, represents 90% of glioblastoma, and is predominantly observed in patients over 55 years old. The progressed from an anaplastic astrocytoma group has mutated IDH and represents 10% of glioblastoma. This group is observed in young patients and its prognosis is easier. The third group is not otherwise specified (NOS) glioblastoma and their status could not be evaluated (Cruz Da Silva et al., 2021). In 2021, the WHO updated glioblastoma classification and introduced new tumor types and subtypes. For the first time, the classification distinctly separates adult- and pediatric-type gliomas, taking into account differences in molecular pathogenesis and prognosis. The 2021 fifth edition of the WHO Classification of Central Nervous System Tumors (WHO CNS5), the significance of laboratory assessments for relevant biomarkers has been heightened for prognostic purposes (Berger et al., 2022). In adults, the classification of diffuse gliomas is streamlined into three types:

1-Astrocytoma, IDH-mutant
2-Oligodendroglioma, IDH-mutant, and 1p/19q-codeleted
3-Glioblastoma, IDH-wildtype (Berger et al., 2022)

In the new update, glioblastomas will now exclusively encompass IDH-wildtype tumors. Mutations in the histone variant 3 (H3) are frequently observed in IDH-wildtype diffuse glioma, especially in pediatric and young adult groups. However, these distinct tumor variants are categorized separately. In IDH-wildtype, H3-wildtype diffuse glioma, the presence of either microvascular proliferation or necrosis is adequate for diagnosing glioblastoma. However, multiple distinctive molecular characteristics are outlined for IDH-wildtype glioblastoma. These include telomerase reverse transcriptase (TERT) promoter mutation, EGFR amplification, and the combined gain of entire chromosome 7 and loss of entire chromosome 10 (+7/−10). These modifications essentially act as criteria for identifying IDH-wildtype glioblastoma. Consequently, any diffuse glioma containing these alterations, even if it presents as grade II or III based on histopathological assessment, is characterized by poor clinical performance (Horbinski et al., 2022; Whitfield and Huse, 2022).

In the updated classification, diffuse astrocytic tumors with IDH mutations are now collectively categorized as “astrocytoma, IDH-mutant” and are given grades II, III, or IV. The grading system incorporates additional molecular markers, such as the presence of a homozygous deletion of CDKN2A/B, which is linked to a poorer prognosis. Specifically, IDH-mutant astrocytomas displaying these molecular alterations are classified as grade IV, regardless microvascular proliferation or necrosis. This refined differentiation between IDH-wildtype and -mutated astrocytomas represents a noteable improvement. However, it places a substantial responsibility on neuropathology laboratories to conduct thorough molecular testing promptly. This is crucial for identifying the 10% of astrocytomas with noncanonical IDH mutations undetectedable using IDH R132H immunohistochemistry and for recognizing astrocytomas with molecular characteristics resembling glioblastoma (Whitfield and Huse, 2022). In the context of pediatric gliomas, they are categorized into low and high grades. Pediatric-type diffuse low-grade gliomas are further divided into four subtypes, while pediatric-type diffuse high-grade gliomas encompass four subtypes. Certain tumor types, like diffuse low-grade glioma and those with MAPK pathway alterations, indicate potential responsiveness to RAF and MEK inhibitors. Additionally, infant-type hemispheric gliomas often feature fusions that could respond to targeted therapies. These classifications and subtypes are intended to offer a more precise comprehension of gliomas and improve treatment strategies (Wen and Packer, 2021).

Monitoring tumor metabolite 2-hydroxyglutarate (2-HG) during surgery offers crucial information such as tumor classification. The presence of 2-HG servesas a guide for optimal resection, while the absence of 2-HG necessitates monitoring other metabolites or lipids. 2-HG-expressing in the central nervous system (CNS) indicates IDH1 or IDH2 mutations (Veliz et al., 2015). The IDH1 mutation remains a robust molecular marker to distinguish these groups. The IDH enzyme, with five isoforms, catalyzes isocitrate to alpha-ketoglutarate (α-KG) and carbon dioxide (CO_2_). Structural alteration due to mutations in IDH1 and IDH2 alter their affinity for isocitrate, leading to the NADPH-dependent reduction of α-KG to 2-HG, resulting in its accumulation in the cells. As an oncometabolite, 2-HG can modify gene expression and inhibit histone demethylation and influence cell differentiation (Turcan et al., 2012; Dang and Su, 2017). Moreover, the primary group can be divided into three subgroups, including 1) metaplastic mesenchymal component of glioblastoma, 2) giant cell glioblastoma, characterized by the presence of multinucleated cells, and 3) epithelioid glioblastoma (Louis et al., 2016).

## 3 Genetic and epigenetic heterogeneity of GBMs

GBMs exhibit genetic and epigenetic heterogeneity, encompassing variations such as amplifications, mutations, and deletions of genes within a tumor. Where cells acquire mutations that are not present in other cells. This genetic heterogeneity results in diverse cell populations with distinct genetic profiles. Epigenetic modifications including histone modifications, DNA methylation, and non-coding RNA molecules can alter gene expression patterns without modifying the underlying DNA sequence. In GBMs, heterogeneity plays a significant role in the development and progression of the tumor (Zhou et al., 2018; DeCordova et al., 2020; Yabo et al., 2022).

Glioma stem-like cells, also known as glioma-initiating cells, are a subpopulation of glioblastoma cells (Wirsching et al., 2016). These cells may originate from the limited population of adult neural stem and progenitor cells found in specific regions such as the subventricular zone, the dentate gyrus of the hippocampus, and the subcortical white matter. Most glioma-initiating cell progenies exhibit features of astrocytes, and some differentiate into functional endothelial cells and pericytes (Wirsching et al., 2016; Gimple et al., 2022).

The gliomas can arise in the glial tissue of the CNS, with occurrencein the astrocytic, oligodendrocytic, or oligoastrocytic tissues (Pan and Monje, 2022). Recent studies have provided evidence that gliomas arise through direct differentiation from progenitor cells, and this process influences the tumor’s response to chemotherapy (Persson et al., 2010). Furthermore, gliomas can be categoriezed based on the degree of invasiveness into two groups: those infiltrating and diffusing into the surrounding brain parenchyma, and frequently recurring after surgical resection, and those with limited growth, manageable through surgical resection (Delgado-M et al., 2020). However, it is important to note that the distinction between glioblastoma classes may not be rigid, with evidence of mosaicism or even class switching observed under the influence of the tumor microenvironment (Veliz et al., 2015).

### 3.1 Genetic heterogeneity

Some of the most common genetic mutations observed in GBMs include:

I. EGFR amplification and mutation, which can result in increased signaling through the phosphatidylinositol 3-kinase (PI3K) pathway and contribute to tumor growth.
II. Loss of heterozygosity (LOH) in chromosome 10, which can result in the loss of tumor suppressor genes such as phosphatase and tensin homolog (PTEN).
III. Mutations in TP53, a tumor suppressor gene that plays a role in regulating the cell cycle and preventing the formation of tumors.
IV. Mutations in the IDH gene, which are more commonly observed in lower-grade gliomas but can also occur in some cases of GBM.
V. Alterations in the retinoblastoma 1 (RB1) gene, which is also involved in regulating the cell cycle.
VI. Mutations in genes involved in the DNA damage response, such as alpha thalassemia/intellectual disability syndrome X-linked (ATRX) (Eskilsson et al., 2018; Hernández Martínez et al., 2022; Verdugo et al., 2022).

Despite sharing identical histology, primary and glioblastoma that originated from a low-grade astrocytoma display distinct differences in their genetic and epigenetic profiles. The primary group is confirmed by amplification and/or mutated EGFR in chromosome 7p, deletion of PTEN, and homozygous deletion of cyclin-dependent kinase inhibitor 2A (CDKN2A-p16^INK4a^) in chromosome 9p. Moreover, in tumors with no TP53 and TERT mutations, amplification of oncogene mouse double minute 2 (MDM2) is observed. NF1 mutations and homozygous deletion of PI3KR1 are also characteristic of this group (Cancer Genome Atlas Research Network, 2008; Vital et al. (2010). The glioblastoma that originated from a low-grade astrocytoma is characterized by the methylation of the promoter of O6-Methylguanine-DNA-methyltransferase (MGMT) associated with TP53 mutations and partial LOH in chromosomes 10q, 13q, 19q, and 22q (Crespo et al., 2015).

Amplification on chromosome 7, deletion on chromosome 10, amplification or mutation in EGFR, and deletion in the locus of Ink4a/ARF define classical glioblastoma. The mutation or deletion in NF1 and expression of Chitinase-3 like-protein-1 (CHI3L1), hepatocyte growth factor receptor (MET), and genes involved in the tumor necrosis factor (TNF) and nuclear factor of *κ*-light polypeptide gene enhancer in B-cells (NF*κ*B) pathways display mesenchymal glioblastoma. Mutation in IDH 1 and 2 is associated with the alterations of PDGFR-α and carries the gliomaCpG island methylator phenotype (GCIMP) is known as proneural glioblastoma (lower-grade gliomas GBM) (Zhu and Wong, 2013). Distinguishing tumors with the glioma-GCIMP phenotype from GCIMP-negative tumors usually have wild-type IDH (Veliz et al., 2015). A review by Mellinghoff et al. (2012) focused on the common genetic alterations observed in growth factor signaling pathways in GBM.

#### 3.1.1 Mutation in EGFR

**FIGURE 1:**
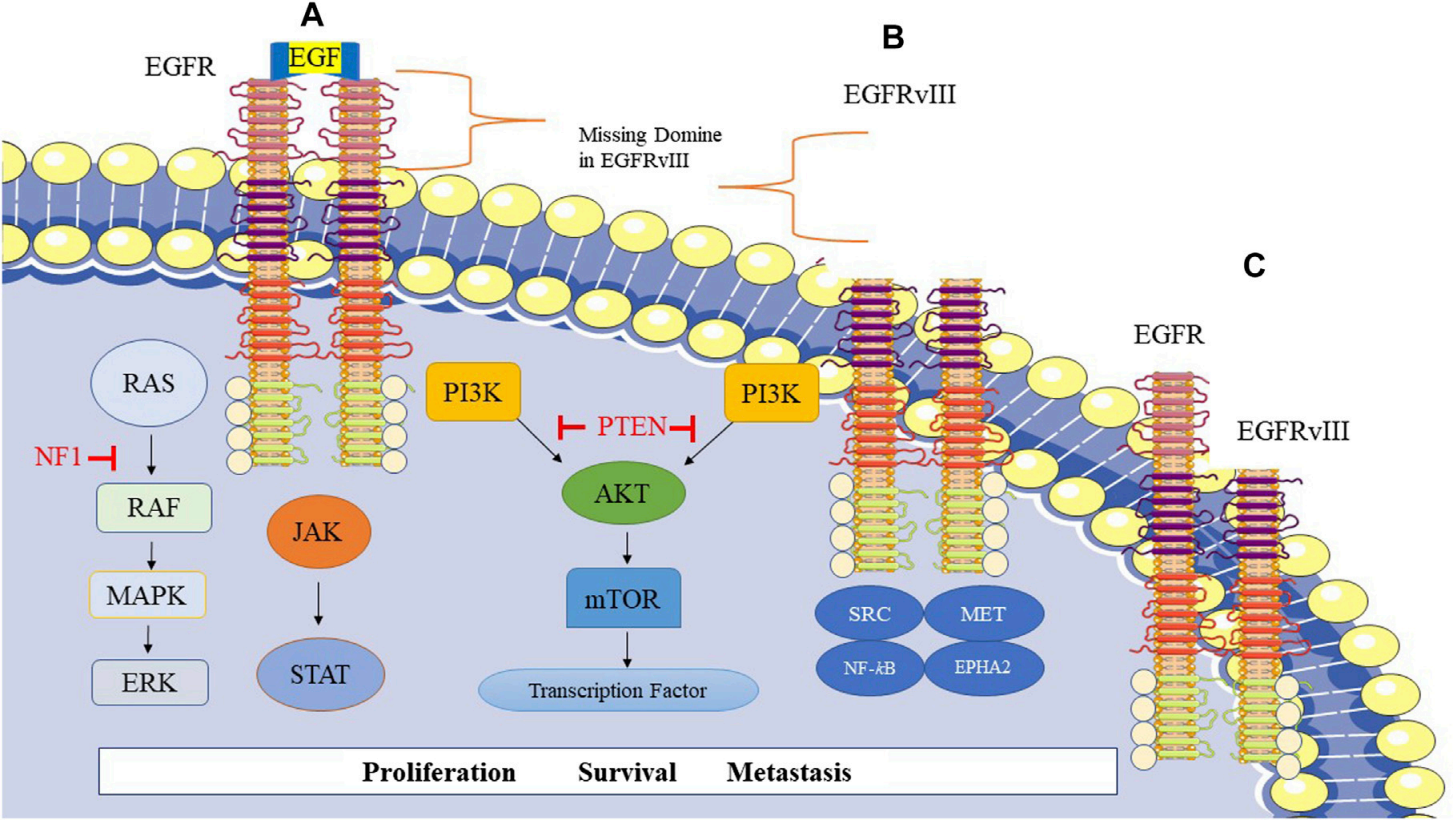
Epidermal growth factor receptor (EGFR) signaling and related pathways in GBM. **(A)** Homodimer of EGFR, **(B)** Homodimer of EGFRvIII, EGFRvIII is a genetic variant of EGFR in glioblastoma cells and frequently occurs in GBM. This mutation leads to the missing extracellular domain in EGFRvIII. **(C)** Heterodimer of EGFR-EGFRvIII.

Mutated EGFR type III (EGFRvIII) is frequently found in approximately 50% of glioblastoma tumors that exhibit EGFR amplification. The deletion in exons 2–7 of the EGFR gene (801 base pairs) generates the EGFRvIII protein, which lacks 267 amino acids in the extracellular domain of EGFR (Figure 1). As a result, this mutated protein cannot bind to ligands and produce a constitutive signal (Gan et al., 2013). However, treatment with EGFR TKIs has shown limited success in glioblastoma compared to lung cancer due to changed kinetics of inhibitor binding or the reduced sensitivity of EGFRvIII (Nishikawa et al., 2004; Bonavia et al., 2012; Vivanco et al., 2012). EGFRvIII requires wild-type EGFR to be an oncogene, as it is activated when wild-type EGFR is co-expressed. EGFRvIII induces the production of heparin-binding epidermal growth factor (HBEGF)-like growth factor, which in turn activates the wild-type EGFR. The activated EGFRvIII may homo- or heterodimerize with EGFR (Figures 1B, C), leading to enhanced transactivation of multiple TK receptor families like MET and EPHA2, mediating EGFRvIII oncogenicity. However, ligands binding to wild-type EGFR can inhibit EGFRvIII and tumor growth (Huang et al., 2007; Veliz et al., 2015).

EGFR/EGFRvIII crosstalk predominantly boosts signal transducer and activator of transcription 3 (STAT3) signaling with less impact on PI3K and mitogen-activated protein kinase (MAPK) signaling pathways. EGFRvIII translocates to the nucleus upon phosphorylation by EGFR, forming a complex with STAT3, resulting in its phosphorylation and activation (Fan et al., 2013).

Francis et al. (2014) demonstrated that multiple EGFR mutational variants exist within glioblastoma tumors, includingEGFRvII and EGFR carboxyl-terminal deletions in the bulk tumor, highlighting the molecular heterogeneity of EGFR alterations in GBM. Therefore, the heterogeneity of glioblastoma is conferred by the plasticity of EGFR amplicons (Francis et al., 2014). The expression of EGFRvII leads to downstream activation of the protein kinase B (AKT) signaling pathway and potentially the STAT3 pathway. Interestingly, EGFR TKI sensitivity is enhanced by EGFRvII. The deletion in exons 2–7 of the EGFR gene generates EGFRvII (Francis et al., 2014; Veliz et al., 2015).

#### 3.1.2 Mutation in PDGFR

PDGF ligands (PDGF-A, PDGF-B, PDGF-C, and PDGF-D) bind to specific receptors on the surface of cells, known as PDGFR (PDGF receptor). PDGFR is a tyrosine kinase receptor, has two isoforms: PDGFR-α and PDGFR-β. Upon binding to PDGF, the dimer of PDGFR-α or PDGFR-β is activated by inducing the receptor dimerization, leading to downstream signaling cascades that trigger cell growth and survival. PDFG and PDFGR are frequently co-expressed in GBM. This co-expression is thought to play a crucial role in the pathogenesis of GBM by promoting the growth and survival of tumor cells. Inhibiting the PDGF/PDGFR signaling pathway has been considered as a promising therapeutic strategy for GBM treatment (Westermark, 2014; Lane et al., 2022). The gene of PDGFR-α is amplified, mutated, or rearranged in GBM. Deletion of exons 8 and 9 in the PDGFR-α gene results in the omission of 243 base pairs and leads to the formation of a constitutively active receptor with tumorigenic ability. Furthermore, a two-base pair deletion in exon 23 can cause truncation of the C-terminal region of the receptor (Mellinghoff et al., 2012; Szerlip et al., 2012).

### 3.2 Epigenetic heterogeneity

GBM is characterized by significant epigenetic heterogeneity with a profound impact on gene expression and cellular phenotype. The phenotypic heterogeneity in GBM is influenced by multiple factors, including the cell of origin and epigenome (Capper et al., 2018). Chromosomal aberrations, such as copy number alterations, can further affect DNA methylation, leading to the formation of epigenetically dynamic regions. DNA methylation profiles can be used to classify GBMs into distinct subclasses that correlate with transcriptomic subtypes (Noushmehr et al., 2010; Chaligne et al., 2021). Additionally, GBMs exhibit heterogeneous DNA methylation and chromatin accessibility profiles not only in different tumor zones but also at the single-cell level, reflecting the diverse phenotypic states of GBM cells (Yabo et al., 2022).

GBMs have been found to co-opt the core transcriptional networks involved in pluripotency reprogramming, similar to those found in embryonic stem cells. Specifically, GBM cells often express high levels of the transcription factors SRY (sex determining region Y)-box 2 (SOX2) and cellular myelocytomatosis (c-Myc), and lower levels of Octamer-binding transcription factor3/4 (OCT3/4), Nanog, and Kruppel-like factor 4 (KIF4) (Rheinbay et al., 2013). Research studies have shown that genetic activation of pluripotency or neural-specific transcription factors [like brain-specific homeobox/POU domain protein 2 (BRN2), Sox2, spalt-like transcription factor 2 (SALL2), and oligodendrocyte transcription factor 2 (OLIGO2)] can induce tumorigenic cancer stem cell-like states in GBM. This is accomplished through modulation of epigenetic regulators, such as the REST corepressor 2/lysine-specific demethylase 1 (RCOR2/LSD1) histone demethylase and DNA methyl transferase Dnmt1, as well as noncoding RNAs such as HOX transcript antisense RNA (HOTAIR) and metastasis associated lung adenocarcinoma transcript 1 (MALAT-1) (Suvà et al., 2014). Sturm et al. (2012) identified three distinct methylation classes in GBM that correlate with patient survival, highlighting the importance of considering epigenetic subtypes in clinical decision-making. Other studies have also identified epigenetic subtypes of GBM that are associated with patient outcomes. Understanding the epigenetic heterogeneity of GBM could pave the way for developing targeted therapies and personalized medicine approaches (DeCordova et al., 2020; Yabo et al., 2022).

## 4 Resistance to temozolomide

The standard treatment for GBM involves a multimodal approach, beginning with surgical resection to remove as much of the tumor as possible. Following surgery, patients typically receive radiotherapy in combination with concomitant adjuvant chemotherapy with temozolomide, a DNA alkylating agent. This treatment is sometimes associated with alternating electric fields of intermediate frequency. Based on chemotherapy-induced disorders, combination therapy may decrease side effects, and increase survival rate (Moslemizad et al., 2022). Generally, recurrence occurs within 12 months of diagnosis in 90% of patients (Li X. et al., 2020; Cruz Da Silva et al., 2021). Temozolomide spontaneously turns to 5-(3-methyltriazen-1-yl) imidazole-4-carboxamide, a reactive methylating agent. This agent then degrades to the methyldiazonium cation, which reacts with DNA and produces DNA methyl adducts such as O6-methyl-guanine, N3-methyladenine, and N7-methylguanine. Consequently, DNA strand breaks occur and cannot be repaired by recombination protein A 51 (RAD51)-driven homologous recombination (HR), resulting in cell-cycle arrest and cell death (Veliz et al., 2015).

Methylation from the O6 position of guanine can be removed by O6-methylguanine-DNA methyltransferase (MGMT), resulting in resistance to temozolomide. In addition, the phosphorylation of STAT3 increases in MGMT-overexpressed glioblastoma cells. It appears that STAT3 is necessary for the posttranscriptional elevation of MGMT. MGMT and phosphorylated-STAT3 levels increase in recurrent tumors compared to primary glioblastoma patients (Kohsaka et al., 2012; Bahadur et al., 2019).

Furthermore, resistance to temozolomide is additionally associated with a deficiency in the mismatch repair (MMR) pathway. MMR is unable to repair the original O6-methyl-guanine lesion. Consequently, impaired MMR function for DNA repair causes breaks in the double strand, replication arrest, and cell death. The failure to recognize this position due to impaired MMR leads to continued DNA replication and resistance to the cytotoxic effect of temozolomide (Hegi et al., 2005; Veliz et al., 2015). Overexpression of base excision repair (BER) contributes to resistance to temozolomide. BER cooperates in the removal of damaged or inappropriate DNA bases such as N7-methyl-guanine. Poly ADP-ribose polymerase (PARP) helps BER and repairs single-stranded DNA breaks. Inhibition of PARP activity induces cell death and enhances cytotoxicity by temozolomide (Veliz et al., 2015).

Chronic exposure to alkylating agents, irradiation, and corticosteroids induces mammalian target of rapamycin (mTOR) expression. mTORC2 transcriptionally and post-transcriptionally modulates N-myc downstream-regulated gene 1 (NDRG1) expression through the serum glucocorticoid-induced protein kinase 1 (SGK1). NDRG1 binds and stabilizes MGMT. Therefore, the mTORC2/SGK1/NDRG1 pathway can be a target for future therapy to overcome glioblastoma resistance (Weiler et al., 2014).

## 5 The kinases signaling pathways

Fleuren et al. (Fleuren et al., 2016) studied the kinome in human cancers, providing crucial information about the dysregulation of the protein kinase superfamily, their role in cancer malignancy, and their sensitivity to anticancer drugs modulated by kinome remodeling (Fleuren et al., 2016). The kinase pathways include receptor and non-receptor TKs activated by phosphorylation in glioblastoma cells. The receptor tyrosine kinases consist of EGFR, erythroblastic oncogene B 2, 3 and 4 (ERBB2, ERBB3 and ERBB4), fibroblast growth factor receptor 3 and 4 (FGFR3 and FGFR4), insulin receptor tyrosine kinase (IRTK), c-rearranged during transfection (c-RET), Insulin-like growth factor 1 receptor (IGF-IR), ephrin type-A receptor 1, 2, 3 and 4 (EPHA1, EPHA2, EPHA3 and EPHA4), macrophage stimulating protein receptor (MSP R), receptor tyrosine kinase like orphan receptor 1 and 2 (ROR1 and ROR2), macrophage colony stimulating factor receptor (M-CSF R), dual leucine zipper kinase (DLK) and tyrosine kinase with immunoglobulin-like and EGF like domains 1 (TIE1). The cytoplasmic non-receptor TKs involve AKT, MAPK, Janus kinase/signal transducers and activators of transcription (JAK-STAT), Wnt/β-catenin, protein kinase A (PKA), cAMP response element-binding protein (CREB), and phospholipase C gamma (PLCɣ) signaling (Joshi et al., 2012). The important signaling pathways that change in glioblastoma include overexpression of EGFR and PDGFR, and activation of Rat sarcoma (RAS), PI3K/PTEN/AKT, RB/CDK N2A-p16^INK4a^, and TP53/MDM2/MDM4/CDKN2A-p14ARF pathways. Moreover, NOTCH signaling is activated and can be linked to hypoxia, PI3K/AKT/mTOR, and ERK/MAPK pathways in grade IV gliomas that increased malignancy (Huse and Holland, 2010; Banerjee et al., 2021). The whole-exome sequencing data demonstrated that at least one receptor tyrosine kinase (RTK) has altered in almost 67% of glioblastoma overall in 291 patients, alteration is EGFR (57%), PDGFRA (13%), c-MET (1.6%), and FGFR (3.2%), also, 25% and 41% of patients have PI3K mutations and PTEN mutations/deletions, respectively (Wang et al., 2021).

### 5.1 EGFR

EGFR is a member of the family of four TKs which includes ErbB1 (EGFR, HER1), ErbB2 (Her-2, Neu), ErbB3 (Her-3), and ErbB4 (Her-4) (Wieduwilt and Moasser, 2008). Mutations and amplifications of EGFR (HER1) have been identified in 45%–57% of studied GBM cases, indicating its potential causal role in GBM pathogenesis. EGFRs are known to promote proliferation and are implicated in both the development of glioblastoma and its resistance to treatment (McLendon et al., 2008; Brennan et al., 2013; Zaki et al., 2021). As discussed above, EGFRvIII and EGFRvII, two truncated mutant forms of EGFR, are expressed in GBM.

Interestingly, ErbB2/HER-2 mutation has also been observed in 8%–41% of GBM cases, indicating that other members of this family may also contribute to GBM development. ErbB2/HER2-specific NK cells can be generated through the isolation of NK cells from peripheral blood donors followed by exposure to ErbB2/HER2 protein or peptides *in vitro*. This exposure leads to the expansion and activation of ErbB2/HER2-specific NK cells, which can be infused into patients with GBM. Promising results have been demonstrated with ErbB2/HER2-specific NK cells in preclinical models of GBM, where they were shown to selectively target and kill glioblastoma cells both *in vivo* and *in vitro* (Zhang et al., 2016; Hosseinalizadeh et al., 2022).

### 5.2 PDGF/PDGFR

The signaling pathway of PDGF/PDGFR is crucial for normal tissue development, but its dysregulation contributes to oncogenesis. The data analyses from the TCGA research network displayed that 10%–13% of the cases studied had amplification of PDGFR-α. The expression of all PDGF ligands (PDGF-A, PDGF-B, PDGF-C, and PDGF-D) and both cell surface receptors, PDGFR-α and PDGFR-β, have been demonstrated in GBM (Pearson and Regad, 2017).

EGFR and PDGFR are RTKs that stimulate signaling pathways. Upon activation, the TK domain of these receptors undergoes autophosphorylation, which leads to the recruitment and activation of PI3K. This, in turn, converts PIP2 to PIP3, which binds to and activates AKT. In the plasma membrane, AKT is phosphorylated at Ser473 and Thr308 by PDK1 and mTORC2, respectively. AKT translocates to the nucleus and activates a cascade of phosphorylation events that ultimately lead to the activation of several proteins involved in angiogenesis, cell growth, and apoptosis, including mTOR and its partner, mTORC1. The tumor suppressor PTEN negatively regulates this pathway by preventing the conversion of PIP2 to PIP3 (Cruz Da Silva et al., 2021). Amplification or activating mutations in EGFR can result in hyperactivation of the PI3K signaling pathway, which promotes tumor growth and survival. In addition, the PI3K pathway can promote lipogenesis through sterol regulatory element-binding protein-1 (SREBP-1) (Veliz et al., 2015).

### 5.3 VEGF/VEGFR

The malignancy of gliomas progresses through angiogenesis. The VEGF and its receptor (VEGFR) are the principal factors of angiogenesis. VEGF is also known to increase the permeability of blood vessels, which allows fluids, nutrients, and other molecules to pass through the walls of the blood vessels more easily (Shibuya, 2011). The upregulation of VEGF promotes angiogenesis to counteract hypoxia, which is a common feature of GBM tumors (Joensuu et al., 2005). Under hypoxic conditions, hypoxia-inducible transcription factors (HIF-1α and HIF-1β) translocate to the nucleus and bind to the hypoxia-response element (HRE) in the promoter region of the VEGF gene, leading to its activation (Lugano et al., 2020). The binding of HIF-1α in the VEGF promoter enhances the angiogenic mechanisms in brain tumors. Furthermore, PDGF, FGF, angiopoietin-1, angiopoietin-2 (ANG-2), delta-like ligand 4 (DLL4), integrins, interleukin-8 (IL-8), and stromal-derived factor 1 (SDF1) besides VEGF can stimulate the angiogenesis in GBM (Delgado-M et al., 2020).

Treatment with nitrosoureas and bevacizumab is used in recurrence of GBM (Delgado-M et al., 2020). Bevacizumab is the monoclonal antibody against VEGFA and targets angiogenesis and was approved for GBM treatment in 2009. Bevacizumab is added to chemoradiotherapy with temozolomide. Gilbert et al. (Gilbert et al., 2014) showed overall survival did not improve when bevacizumab was used in patients with newly diagnosed glioblastoma. Additionally, in a study by Chinot et al. (Chinot et al., 2014) was demonstrated that bevacizumab addition to radiotherapy and temozolomide did not improve overall survival, Moreover, the use of bevacizumab was associated with a higher rate of adverse effects compared to placebo.

### 5.4 RAS/MAP/ERK signaling pathway

Many studies have reported that 88% of GBMs have mutations in RAS/MAPK and PI3K/AKT pathways which play the principal role in multiple cellular processes. The RAS/MAP/ERK pathway is a vital signaling pathway that modulates cell growth, differentiation, and survival. Mutations or dysregulation in this pathway can cause abnormal activation and lead to uncontrolled cell proliferation, tumorigenesis, and metastasis in various cancers (Pearson and Regad, 2017).

The pathway is initiated by the activation of RAS proteins, which are localized on the cell membrane. Activation of the RAS/MAPK causes GDP transformation to GTP, RAS undergoes a conformational change that leads to interact with downstream signaling molecules (Regad, 2015). RAS activates rapidly accelerated fibrosarcoma (RAF), which in turn activates MEK and ultimately results in the activation (phosphorylation) of ERK. ERK translocates to the nucleus where it modulates gene expression, thereby regulating various cellular processes such as cell growth, differentiation, and survival (Kolch, 2000; McCubrey et al., 2007). Hyperactivation of this pathway increases growth autonomy and glioblastoma migration (Pearson and Regad, 2017). Additionally, the pathway has also been associated with the development of resistance to chemotherapy and radiation therapy. Therefore, understanding the RAS/MAPK pathway’s mechanisms and identifying its aberrations is critical for developing targeted therapies and improving cancer treatment outcomes (McCubrey et al., 2007).

Astrocyte elevated gene-1 (AEG1) as a target of RAS activates multiple signaling pathways such as PI3K-AKT, MAP/ERK, Wnt, and NF*κ*B. In addition, the expression of AEG1 has a negative correlation with the excitatory amino acid transporter 2 (EAAT2). The suppression of EAAT2 results in a reduction of glutamate uptake by glial cells (Berger et al., 2022).

### 5.5 Other tyrosine kinase pathways

The aberrant activation of NF*κ*B is observed in GBM, making it an attractive target for cancer prevention or treatment (Ghareghomi et al., 2021). This abnormal activation of NF*κ*B is thought to contribute to the development and progression of glioblastoma by promoting cell proliferation, inhibiting cell death, and promoting inflammation. The EGFR pathway activates the transcription factor NF*κ*B (Soubannier and Stifani, 2017). Bredel et al. (Bredel et al., 2011) showed that NFKBIA (nuclear factor of κ-light polypeptide gene enhancer in B-cells inhibitor-α) deletion and EGFR amplification have a similar effect in the pathogenicity of GBM, but their effect is exclusive. NFKBIA is an inhibitor of NF*κ*B and suppresses glioblastoma tumors. Loss of NFKBIA function results in NF*κ*B activation, which contributes to glioblastoma progression. On the other hand, EGFR amplification leads to increased signaling through the PI3K/AKT and MAPK pathways, promoting cell growth and survival (Bredel et al., 2011).

Activation of hepatocyte growth factor receptor (HGFR) also known as c-MET can occur through several mechanisms such as gene amplification, mutation, or ligand binding, and plays a role in cell proliferation, differentiation, and migration. HGF as a ligand binds to c-MET on the surface of tumor cells and can lead to downstream signaling pathways. Dysregulation of the c-MET pathway has been implicated in the pathogenesis of glioblastoma. Overexpression of c-MET has been observed in GBM and is linked to poor prognosis (Kong et al., 2009; Petterson et al., 2015).

The overexpression and amplification of FGFR genes is observed in GBM which causes the activation of FGFR signaling and leads to enhanced tumor growth and invasion. In addition, FGFR signaling has been shown to promote the maintenance of glioblastoma stem cells, which are thought to be responsible for tumor recurrence (Loilome et al., 2009).

The urokinase plasminogen activator (uPA) and its receptor (uPAR) are frequently upregulated in GBM, leading to increased activation of plasminogen and promoting tumor cell migration and invasion. The PI3K/AKT signaling pathway appears to be involved in regulating uPA-induced cell migration, as inhibition of this pathway can downregulate uPA activity. Additionally, uPA can activate matrix metalloproteinases (MMPs), which further contribute to the invasive phenotype of glioblastoma cells (Delgado-M et al., 2020).

Cell motility in glioma cells is associated with Rho-family GTPases, including RhoA, Ras-related C3 botulinum toxin substrate (RAC), and cell division control protein 42 homolog (CDC42), which regulate the actin cytoskeleton. The myosin-actin interactions are promoted through Rho-associated coiled-coil kinase (ROCK), while the formation of lamellipodia is activated by Rac and the formation of filopodia is activated by CDC42. Dysregulation of Rho-family GTPases is observed in glioblastoma and contributes to increased cell motility, invasiveness, and tumor progression (Delgado-M et al., 2020).

The SRC family tyrosine kinases (SFKs) belong to the broad family of non-receptor tyrosine kinases. SRC can regulate the PI3K/AKT/mTOR axis which suppresses autophagy. SRC activity is overexpressed in GBM. Inhibition of SRC tyrosine kinase can induce autophagy in GBM (Jovanović Stojanov et al., 2022).

In 2008, the TCGA suggested that dysregulation in the RB, p53, and RTK/RAS/PI3K pathways are obligatory events in glioblastoma tumors and can help guide therapeutic decisions. Treatment with cyclin-dependent kinases (CDK) inhibitors can be expected in patients with amplifications of CDK4/CDK6 or inactivating mutations or deletions in CDKN2A or CDKN2C. Furthermore, PI3K or PDK1 inhibitors might be effective for patients with PTEN deletions or activating mutations in PIK3CA or PIK3R, whereas the PI3K pathway that is altered by AKT3 amplification is resistant. Therefore, the design of RTK inhibitors cocktails might be a beneficial strategy to treat the multiple phosphorylated (activated) RTKs in individual glioblastoma specimens (McLendon et al., 2008).

## 6 Tyrosine kinase inhibitors (TKIs)

The Sokolov et. al. (Sokolov et al., 2021) study found that kinase inhibitors are a versatile class of drug targets in clinical trials for brain cancers, with 87 unique proteins of kinases. These included isoforms of several kinases for the PI3K-AKT-MTOR pathway, Janus kinase (JAK), EGFR, ERBB2, FGFR, anaplastic lymphoma kinase, KIT, cyclin-dependent kinases (CDKs), mitogen-activating protein kinase, tyrosine-protein kinase Lyn, tropomyosin receptor kinase, EPHA2, WEE1 kinase, and many other targets. The meta-analysis showed that anti-EGFR therapies have no impressive effects on the overall survival of patients with GBM (Lee et al., 2020). The inhibitors of angiogenesis have often been combined with other therapies, and a few combinations with bevacizumab have only reached phase III of clinical trials (Sokolov et al., 2021). According to the meta-analysis conducted by Ameratunga *et al.* (Ameratunga et al., 2018), antiangiogenic treatment did not provide any improvement in overall survival for patients with high-grade GBM.

Cabozantinib targets several RTKs, including VEGF/VEGFRs, MET, and AXL. VEGFR and MET are known to promote tumor growth and metastasis by regulating angiogenesis, cell proliferation, cell migration, and epithelial-to-mesenchymal transition. AXL kinase, on the other hand, is implicated in tumor pathogenesis and signaling pathways that promote metastasis. By targeting these RTKs, cabozantinib has shown promise in the treatment of several types of cancer, including renal cell carcinoma, hepatocellular carcinoma, and medullary thyroid cancer (Maroto et al., 2022). Cabozantinib shows *in vivo* efficacy in multiple xenograft models. It has also demonstrated synergistic effects with radiation therapy in glioblastoma cell lines. A phase II clinical trial evaluated the safety and efficacy of cabozantinib in patients with recurrent glioblastoma (Wen et al., 2018).

Sorafenib inhibits RAF, PDGFR, VEGFR, c-KIT, and FLT3. However, this multitarget TKI failed in phase III of the clinical trial (Wilhelm et al., 2008).

Joshi et al. (Joshi et al., 2012) reported that the combination of gefitinib and sunitinib, as well as sunitinib and sorafenib, can inhibit the phosphorylation of MAPK, AKT, and STAT3. The gefitinib and sunitinib combination was found to decrease the phosphorylation of several TKs, including EGFR, FGFR3, ERBB2, MER, TIE2, INSULIN R, rearranged during transfection kinase (C-RET), DLK, TIE1, EPHA1, EPHA4, AKT, MAPK, PKA (CREB), SRC, JAK-STAT, c-JUN, and p53. Therefore, targeting multiple TKs in combination therapy might be an effective approach. However, this combination did not demonstrate any survival benefit in animal models. The authors suggested that targeting multiple targets and improving the drug delivery system should be considered for a successful therapeutic strategy.

Manzano et al. (Manzano et al., 2021a) have demonstrated that patients with GBM who have low C3G expression may not respond to EGFR inhibitors. The downregulation of C3G results in the reduction of EGFR levels. C3G is a guanine nucleotide exchange factor (GEF) for GTPases from the RAS superfamily and can also act through GEF-independent mechanisms. C3G can modulate RTKs such as EGFR, tyrosine kinase receptor A (TRKA), anaplastic lymphoma kinase (ALK), MET, and IRTK, and stimulate proliferation and differentiation in neural cells. It appears that C3G (RAPGEF1) mRNA levels are downregulated during the onset and progression of GBM. However, using C3G as a target for GBM treatment is still not recommended (Manzano et al., 2021a; Manzano et al., 2021b).

Everolimus, an mTOR inhibitor, has received approval for the treatment of subependymal giant cell astrocytoma (SEGA), and is being investigated in combination with other drugs such as temozolomide, lenvatinib (a VEGFR inhibitor), sorafenib, ribociclib (a CDK inhibitor), and dasatinib (a BCR/ABL and SRC inhibitor). Despite the variety of kinase inhibitors available, selumetinib, a mitogen-activated protein kinase 1/2 inhibitor, has successfully passed phase III trials in low-grade glioma and astrocytoma (NCT03871257, NCT04166409) (Sokolov et al., 2021).

Despite advanced knowledge in molecular biology and genetics of GBM due to its heterogeneity, developing an effective therapy is an obstacle. In GBM drug design, permeability and pharmacokinetics should be considered due to the impermeable BBB (Mitusova et al., 2022). For example, gefitinib and erlotinib are EGFR inhibitors that have failed in GBM treatment due to their inability to effectively penetrate the BBB, which limits their concentration in the brain (Pan et al., 2020).

## 7 Challenges in developing selective TKIs

As mentioned, gefitinib, erlotinib, lapatinib, dacomitinib, and osimertinib are EGFR inhibitors received approval for non–small cell lung cancer (NSCLC) treatment. The results revealed pharmacokinetic failure in GMB therapy is related to BBB penetration of these inhibitors (Wang et al., 2021). Hence, some improvements to this kind of inhibitor are being developed, like the combination of AZD3759, a blood-brain barrier-penetrant EGFR inhibitor, and WSD0922, a selective EGFR exon 20 insertion mutant inhibitor, which is promising to evaluate the role of EGFR signaling inhibition. Epitinib and AZD3759 are in clinical trials for untreated EGFR-mutant NSCLC with brain metastases and have shown efficacy in patients (Zeng et al., 2015; Zhou et al., 2022). Furthermore, dacomitinib has shown promising results in early-phase clinical trials for patients with recurrent glioblastoma who have EGFR amplification, with or without EGFRvIII. Further clinical trials are required to evaluate its efficacy and safety in a broader patient population (Sepúlveda et al., 2014).

Clinical trials testing anti-angiogenic agents such as bevacizumab, PDGFR, VEGFR, and PKC inhibitors, have not demonstrated significant improvements in overall or progression-free survival compared to standard therapy (Schulte et al., 2021). Rapid resistance development and the potential contribution of factors beyond angiogenesis, such as invasion and immune evasion, may underlie the limited efficacy of these agents in treating glioblastoma (Voutouri et al., 2019).

Indeed, Dasatinib is a multi-kinase inhibitor that targets various kinases, such as SRC, PDGFR, KIT, EPHA2, and BCR-ABL fusion. However, its effectiveness in treating brain tumors is hampered by its inadequate accumulation in the brain. This limitation arises from the activity of P-glycoprotein and related molecules, which actively transport drugs out of the brain, thereby reducing their concentration within the target area (Lassman et al., 2015; Palande et al., 2022). Several clinical trials with EGFR inhibitors have failed because of low CNS penetrance, tumor heterogeneity, and pharmacokinetics properties (Wen et al., 2014; Wen et al., 2020). Despite more than 15% of clinical trials focusing on brain cancer, it is surprising that kinase inhibitors have not achieved treatment success. A significant challenge in developing drugs for brain cancer lies in pharmacokinetic properties, primarily due to BBB, which restricts the passage of molecules exceeding 500 Da, especially those that are lipid-insoluble and polar. Therefore, small molecules are the best candidates for TKIs. However, the desired distribution requires delivery systems. Various delivery systems such as liposomes, polymer nanoparticles, metal nanoparticles, bacterial-derived carriers, and protein nanoparticles have been developed for this purpose (Sokolov et al., 2021). The development of GBM occurs in the interstitial space of the brain, which is separated from the systemic circulation by the BBB. The tumor growth and angiogenesis lead to changes in the function and permeability of the BBB, which can affect the delivery of drugs to the tumor site. The expression of aquaporin proteins, which are involved in water transport across the BBB, can also change during glioblastoma development and contribute to BBB dysfunction (Silantyev et al., 2019).

### 7.1 The blood-brain barrier (BBB)

The blood-brain barrier is a neuroprotective barrier comprised of a monolayer of endothelial cells, along with ependymal and tanycytic cells. These cells are tightly interconnected by adherens junctions and tight junctions, which effectively restrict the passage of harmful substances into the brain. Occludin, claudin, and junction adhesion molecules are the chief proteins of tight junctions. Serine, threonine, and tyrosine phosphorylation regulate occludin (OCLN). The formation of tight junctions during the acquisition of cell polarity is regulated by junction adhesion molecules. Additionally, zonula occludens and cingulin also help the maintenance and integrity of BBB (Daneman and Prat, 2015; Kadry et al., 2020).

Furthermore, the development, function, and maintenance of the BBB are closely associated with the endothelium and related to nerve terminals, astrocytes, pericytes, CNS-border associated macrophages (BAMs), and a specific myeloid subpopulation. Moreover, the blood-brain tumor barrier (BBTB) also inhibits the entrance of drugs to the tumor bulk. The density of the endothelial cell layer in the BBB is not compromised during alterations at the tumor site; therefore, the function of the BBB remains efficient (Banerjee et al., 2021). The accumulation of small molecules, including potential therapeutics, can be limited by efflux pumps such as P-glycoprotein (P-gp, ABCB1), breast cancer resistance protein (BCRP, ABCG2), and multidrug resistance-associated proteins (MRP1, 4 and 5, ABCC1, 4 and 5), which are members of the ATP-binding cassette (ABC) transporter superfamily (Sarkar et al., 2018; Mo et al., 2021).

In GBM, the BBB is disrupted due to the infiltration of tumor cells and the secretion of various factors, such as VEGF, that promote angiogenesis and BBB leakage. This disruption can lead to increased permeability of the BBB, allowing for the entry of circulating cells and molecules into the brain. At the same time, the BBB in the peritumoral region may remain intact, creating a BBTB that limits drug delivery to the tumor. Therefore, strategies to target both the BBB and BBTB are being developed to improve drug delivery and treatment efficacy in GBM (Lugano et al., 2020).

The small molecules transport in and out of the brain by active transport, endocytosis, carrier-mediated transport, and passive diffusion (Chowdhury et al., 2021). Several challenges exist for drug transportation in GBM due to neovascular complexity, including effective permeation and drug concentration in brain cells. Efflux pumps recognize and eliminate foreign substances on the brain’s luminal side, and ABC transporters can act as obstacles to drug entry into the brain. Furthermore, uptake and efflux transporters can become saturated when exposed to inhibitory signals. Although tumors compromise the structural integrity of the BBB and make it leaky to small molecules at the tumor site, the BBB remains intact at the tumor’s edge, which is surrounded by proliferating cells (Gomez-Zepeda et al., 2019; Patel et al., 2021).

Scientists at Pfizer have developed a novel algorithm called CNS multiparameter optimization (CNS MPO) to address some of the challenges in drug discovery for brain targets. This algorithm consists of physicochemical parameters: ClogP (lipophilicity, calculated partition coefficient), ClogD (calculated distribution coefficient at pH 7.4), MW (molecular weight), TPSA (topological polar surface area), pKa (most basic center), and HBD (the number of hydrogen bond donors) with a score of 0 for low probability and 1 for high probability. Thus, the summation of the scores is between 0 and 6. The study by Wager et al. (Wager et al., 2010) reported that a high score in the CNS MPO algorithm was associated with a higher probability of a compound being a successful CNS drug, as evidenced by the fact that 74% of marketed CNS drugs have a score of four or more. Shergalis et al. (Shergalis et al., 2018) identified 73 potential drug candidates for GBM and found that only 37% of the small molecule candidates had a score of more than four in current clinical trials, indicating that the majority of these candidates may not have favorable physicochemical properties for effective CNS drug delivery. Therefore, this algorithm, accompanied by other available tools, can be used by medicinal chemists to expedite the identification of compounds with an enhanced probability of success at the design stage.

#### 7.1.1 Limited brain penetration of TKIs

The drug candidate must have proper pharmacokinetics properties, like reaching therapeutic concentrations at the tumor site without diffusing into other tissue (Sun et al., 2022). Erlotinib and gefitinib efficacy is limited and efflux transporters such as P-gp and ABCG2 remove the drugs from the brain. Gefitinib is only effective in patients whose tumors have specific mutations in exons 19 and 21 of the EGFR domain (Agarwal et al., 2010; Lo, 2010; de Vries et al., 2012; Tournier et al., 2021). Osimertinib and afatinib, are substrates of P-gp, and hence, are effluxed back to the bloodstream (Wind et al., 2014; van Hoppe et al., 2019). Additionally, neratinib, a pan-EGFR inhibitor, is a substrate for P-gp and ABCG2 and has limited brain penetration. A pan-EGFR inhibitor is a type of drug that inhibits all members of the epidermal growth factor receptor family, which includes HER1, HER2, HER3, and HER4 (Feldinger and Kong, 2015). Furthermore, lapatinib, a dual HER1/HER2 inhibitor cannot efficiently cross the BBB (Higa and Abraham, 2007).

Perifosine is an inhibitor of AKT signaling, which is a key pathway involved in the growth and survival of cancer cells. However, preclinical studies have shown that perifosine has limited brain penetration, which could limit its effectiveness in treating brain tumors (Cole et al., 2015; Becher et al., 2017).

Foretinib and SGX523 are two inhibitors for c-MET but the data that show their penetration into the brain is inadequate. Significant side effects were observed for cabozantinib, which can inhibit c-MET and VEGFR2. However, the selective MET inhibitor, capmatinib (INC280), is under GBM clinical evaluation (NCT02386826) (Zhang et al., 2010).

Heffron et al. (Heffron, 2016) reported that many small molecule inhibitors designed to target VEGFR/PDGFR have limited brain penetration due to their substrate nature for efflux transporters such as P-gp and BCRP. Cediranib, pazopanib, sunitinib, sorafenib, regorafenib, tandutinib, axitinib, and vatalanib are examples of such inhibitors. However, cabozantinib and brivanib have been reported to exhibit minimal P-gp mediated efflux and could be potential targets for GBM treatment.

GDC-0084, pilaralisib, buparlisib, XL765, and PX-866 are PI3K/mTOR inhibitors that can cross the BBB leading to their advancement to clinical trials for the treatment of GBM (Zhao et al., 2017; Colardo et al., 2021). Unfortunately, buparlisib has been reported to induce mood changes (Wright et al., 2021). Everolimus and sirolimus are FDA-approved agents that inhibit mTORC1 but are substrates of P-gp. In addition, perifosine inhibits AKT signaling but has brain penetration limitation preclinically (Heffron, 2016).

Palbociclib and abemaciclib are CDK4 and CDK6 inhibitors that are substrates of both P-gp and BCRP (Groenland et al., 2020). *In vitro* studies have revealed that CDK1 and 2 inhibitors such as flavopiridol, seliciclib, dinaciclib, SNS-032, and AT7519 are still being evaluated through clinical trials and further research (Gojo et al., 2013; Sánchez-Martínez et al., 2015; Dichiara et al., 2017).

Additionally, imatinib, cediranib, pazopanib sunitinib, sorafenib, tivozanib, nintedanib, and dovitinib inhibit PDGF receptors but did not show a survival benefit due to poor BBB penetration (Wang et al., 2021). Crenolanib has been investigated in a phase II clinical trial (NCT02626364) involving GBM patients with PDGFRA gene amplification. This inhibitor selectively inhibits the signaling of wild-type and mutant isoforms of the PDGFR family. Crenolanib effectively inhibits phosphorylation of PDGFR-α and downstream AKT signaling in Ink4a/Arf^−/−^. However, further research is needed to fully understand the potential of crenolanib and other PDGFR inhibitors in treating GBM (Paugh et al., 2013).

The modifications in the structure of gefitinib have been made to improve its physical properties and reduce transporter-mediated efflux. Similarly, AZD3759 (third-generation of TKIs), a pan-EGFR inhibitor, has been developed with reduced rotatable bonds and sufficient hydrogen bond donors, allowing it to cross the BBB more easily than gefitinib. Tucatinib, the inhibitor for phospho-HER2, was reported to be able to cross the BBB freely. Several clinical trials evaluating tucatinib have been completed or are currently ongoing (Borges et al., 2018; Kulukian et al., 2020). The third-generation EGFR inhibitor osimertinib (AZD9291) and GDC-0084 have demonstrated greater permeability in a Phase I dose-escalation study conducted in patients with high-grade GBM (Ballard et al., 2016; Wen et al., 2020).

### 7.2 pharmacokinetic and pharmacodynamic properties of TKIs

Although TKIs share similar mechanisms of action, they vary in their ability to target specific kinase profiles, pharmacokinetic properties, and potential side effects. Hartmann et al*.* (Hartmann et al., 2009) summarized the pharmacology, metabolism, and side effects of TKIs. TKIs are designed to bind to the ATP-binding site of the tyrosine kinase, thereby preventing ATP from binding and inhibiting the kinase activity. Most kinase inhibitors exhibit ATP-competitive binding, which is attributed to the presence of a large hydrophobic surface in the ATP binding pocket. This feature enables these inhibitors to bind with high affinity to the kinase, as they can effectively interact with the hydrophobic environment of the pocket (Knight and Shokat, 2005). While the exact structure of small TKIs can vary depending on the specific compound, there are some common features and structural motifs found in many TKIs, which are documented in databases such as PubChem and ChemSpider. The prevalent structure includes a core scaffold consisting of a central aromatic ring system or a heterocyclic ring, an ATP-mimetic moiety often including a substituted purine or pyrimidine ring, binding interactions that can involve hydrogen bonding, hydrophobic interactions, electrostatic interactions, and Van der Waals forces, as well as substituents that can influence their potency, selectivity, and pharmacokinetic properties. Additionally, TKIs exhibit variability, as different compounds are designed to target specific TKs or address specific disease indications (Roskoski, 2019). A new generation of allosteric kinase inhibitors has been discovered. These inhibitors target allosteric sites on kinases, providing a different approach compared to traditional ATP-competitive inhibitors. This allosteric targeting offers a promising strategy for developing highly selective and potent kinase inhibitors, which may lead to improved therapeutic outcomes.

**FIGURE 2:**
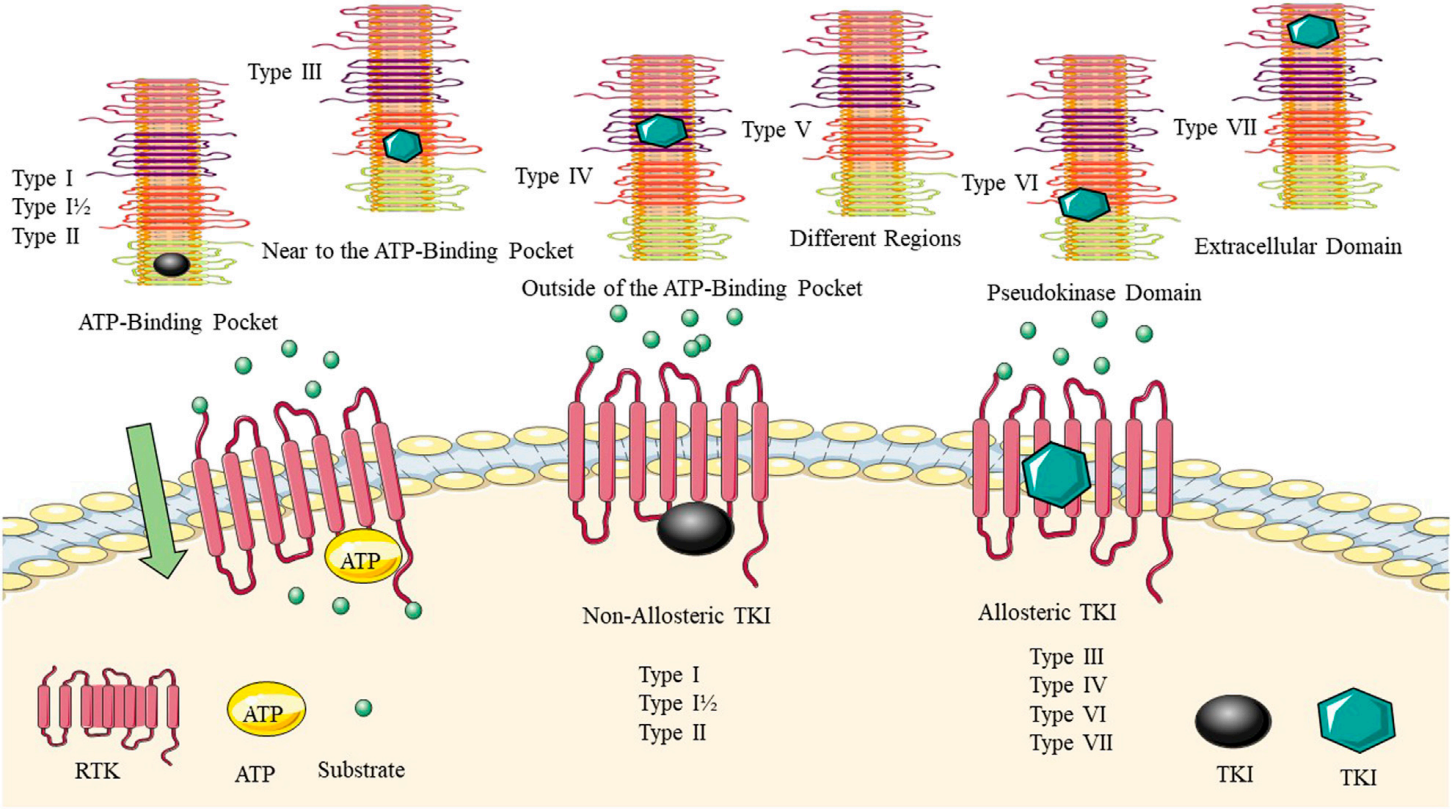
Allosteric and non-allosteric tyrosine kinase inhibitors. The binding site of the TKI for each group is highlighted. Type I–V inhibitors are reversible. Type VI kinase inhibitors can form covalent bonds with kinase sites, leading to the irreversible alteration of target activity.

Bhullar et al. (Bhullar et al., 2018) described the types of allosteric and non-allosteric inhibitors of TKs (Figure 2). Allosteric inhibitors bind to a site that is distinct from the ATP-binding pocket, called the allosteric site, and can induce conformational changes that inhibit kinase activity. Non-allosteric inhibitors, bind to the ATP-binding site and compete with ATP for binding to the kinase. Hence, Type I, such as cabozantinib and gefitinib, compete and bind to the ATP-binding pocket of the active conformation of proteins. In contrast, type II kinase inhibitors, including sorafenib, imatinib, and nilotinib, bind to the inactive conformation of protein kinases. While the binding sites of type III and IV are not located in the ATP pocket and function through allosteric mechanisms, only a few TKIs of these types, such as asciminib, have been approved. The type I–V inhibitors are reversible. Type VI kinase inhibitors can form covalent bonds with kinase sites, leading to the irreversible alteration of target activity. Osimertinib, afatinib, and ibrutinib possess better pharmacokinetic properties than reversible inhibitors (Hartmann et al., 2009; Lu et al., 2020).

Classifying ATP-competitive kinase inhibitors presents a challenge due to the variability in their molecular structures and the complexity of the conformational space occupied by kinase-inhibitor complexes. Inhibitors can bind to multiple conformational states of the kinase, making the classification process even more complicated (Arter et al., 2022). Robert Roskoski (Roskoski, 2023) described how small molecule protein kinase inhibitors can be classified into seven main groups based on their mechanism of action. The groups include reversible inhibitors (Groups I, I½, II, III, IV, and V) and targeted covalent irreversible inhibitors (VI). The type I½ and type II inhibitors are further divided into A and B subtypes, with subtype A inhibitors extending past the gatekeeper residue into the back cleft, while subtype B inhibitors do not. It is suggested that subtype A inhibitors may bind to their enzyme target with longer residence times compared to subtype B inhibitors. The example of sorafenib and sunitinib is given, with sorafenib being a type IIA VEGFR blocker with a residence time exceeding 64 min and sunitinib being a type IIB VEGFR inhibitor with a residence time of less than 2.9 min. Overall, the classification of small molecule protein kinase inhibitors into these groups and subtypes can aid in understanding their mechanisms of action and potential therapeutic benefits (Roskoski, 2023).

TKI resistance is a major challenge that significantly reduces patients’ survival and quality of life. The abnormal activation of protein kinase-related signaling pathways due to gene mutations is the main reason for TKI resistance, and the tumor microenvironment also plays a crucial role. Cell death resistance, immune reprogramming, tumor metabolism, and epigenetic modifications are other mechanisms involved in TKI resistance (Yang et al., 2022). Therefore, due to the heterogeneity of TKI resistance mechanisms, a single therapeutic strategy may not be effective in all patients, and a deeper understanding of the mechanisms is essential.

## 8 Rational drug design of TKIs by computer-aided

The binding pockets found in kinase proteins are highly similar in structure, making it challenging to develop inhibitors that specifically target one particular kinase and can contribute to adverse effects (Ravikumar et al., 2019). Various methods have been developed over the years to improve kinase selectivity. The first generation of TKIs was developed as ATP-competitive inhibitors. Second-generation TKIs were developed as allosteric inhibitors. Third-generation TKIs have been developed to address resistance mutations that occur during treatment with first- and second-generation TKIs. These mutations can occur in the kinase domain and lead to structural changes that hinder the binding of earlier TKIs. By selectively binding to the mutant kinases, these inhibitors aim to restore the efficacy of kinase inhibition and improve treatment outcomes (Huang et al., 2020; Kim and Ko, 2020; Hirschbühl et al., 2021).

Bioinformatics plays a pivotal role across various stages of the drug design process, including lead compound screening, target protein discovery, understanding the mechanism of drug action, and clinical statistical analysis (Li K. et al., 2020). Bioinformatics facilitates the identification of molecules with specific chemical structures for desired pharmacological effects in lead compound screening and, for target protein discovery, involves analyzing known effective target genes by quantifying their characteristics and comparing homologies with potential new target genes (Behl et al., 2021). In addition, bioinformatics plays a crucial role in drug development by assessing target druggability to reduce project failure risks, examining the similarity between different drugs to enhance understanding of drug mechanisms, utilizing clinical statistical analysis to evaluate the clinical effectiveness of compounds, and employing computational techniques to explore drug-target interactions and the role of proteins in drug mechanisms (Woolle et al., 2017; Wang et al., 2023).

Rational drug design, also known as computer-aided drug design (CADD), is a powerful tool used in the development of TKIs. CADD allows researchers to use computer simulations and modeling to predict how drug molecules interact with their targets and optimize the drug’s properties such as selectivity, affinity, and pharmacokinetics (Yu and MacKerell, 2017). One approach to rational drug design is to use the crystal structures of protein kinases to design inhibitors that fit into the active site of the kinase. By using computational modeling and molecular dynamics simulations, researchers can predict which compounds are likely to bind with high affinity to the kinase and selectively inhibit its activity and named as structure-based drug design (SBDD) (Prieto-Martínez et al., 2019). Another approach is to use virtual screening methods to identify potential kinase inhibitors from large compound libraries, similarity searching, quantitative structure-activity relationship (QSAR) modeling, and pharmacophore generation which is named ligand-based drug design (LBDD) (Giord et al., 2022). Gagic et al. (Gagic et al., 2020) reviewed the CADD methods for the design of TKIs as anticancer drugs. The authors also provided examples of how to design new inhibitors for specific targets such as EGFR, VEGFR, PI3K, and MAPK (Gagic et al., 2020). Furthermore, several databases provide information on TKIs like ChEMBL (Gaulton et al., 2017), Kinase Knowledgebase (KKB), (Sharma et al., 2016), Protein Kinase Inhibitor Database (PKIDB) (Carles et al., 2018), and BindingDB (Gilson et al., 2016) that can be helpful for researchers to search for potential protein kinase inhibitors and their properties, as well as to analyze the structure-activity relationships of known inhibitors.

## 9 Future direction

The emergence of multi-omics data facilitates computational predictions for anticancer drugs by revealing potential repositioning opportunities. To address the complexity of patient responses in cancer treatment, bioinformatics methods leverage patient-specific genetic, epigenetic, metabolomic, and transcriptomic profiles for precise drug selection, ultimately improving clinical outcomes. Omics technologies play a crucial role in unraveling the mechanisms of cancer progression and identifying biomarkers and treatment targets (Baysoy et al., 2023). Large-scale initiatives, such as the Pan-Cancer Analysis of Whole Genomes (PCAWG) Consortium, have generated extensive omics data, enabling advanced studies on gene mutations and expression profiles across diverse cancers. Notable datasets, including the NCI-60 Human Tumor Cell Lines Screen, Genomics of Drug Sensitivity in Cancer (GDSC), The Cancer Genome Atlas (TCGA), Cancer Therapeutic Response Portal (CTRP), L1000 profiles from The Library of Integrated Network-Based Cellular Signatures (LINCS) Program, Cancer Cell Line Encyclopedia (CCLE), and the Catalogue of Somatic Mutations In Cancer (COSMIC), have proven valuable in understanding drug-resistant cancer cells. These datasets provide novel insights, and the increasing volume is expected to drive the development of computational models that systematize approaches to studying drug-resistant cancer cells more effectively (Nicora et al., 2020; Cai et al., 2022). Particularly, the integration of multi-omics analyses with advanced tools like genome engineering like CRISPR-Cas9 will remain pivotal for the comprehensive characterization of drug-resistant cancer cells. The growing abundance of omics data is expected to contribute to the development of diverse computational models. Consequently, the outcomes predicted by these models will enable a more systematic design of experiments focused on drug-resistant cancer cells (Jung et al., 2021). In recent years, machine learning (ML) and artificial intelligence (AI) have also been applied to the rational drug design of TKIs. These methods can rapidly process large amounts of data and generate predictive models that can guide the design of novel inhibitors with improved properties (Urbina et al., 2021; Moriwaki et al., 2022; Bao et al., 2023).

## 10 Conclusion

GBM is characterized by high molecular and transcriptional heterogeneity, which contributes to therapy resistance. Despite recent advancements in targeted therapies, particularly TKIs against GBM, their success has been limited. This is primarily due to their poor penetration of the BBB and inadequate achievement of pharmacokinetic concentrations. Additionally, resistance to TKIs poses a significant challenge in cancer treatment, especially with long-term use. Resistance can arise from genetic alterations, alternative signaling pathways, or changes in the tumor microenvironment. Understanding the mechanisms of resistance and developing new strategies to overcome it is crucial for enhancing the efficacy of TKIs in cancer treatment.

To address this, several reliable methodologies have been developed to profile kinome activity by monitoring substrate or kinase phosphorylation in a high-throughput manner. These techniques have greatly contributed to our understanding of biological and pathological processes, enabling the identification of key kinases involved in disease progression. Such approaches play a vital role in discovering druggable targets and provide valuable insights into potential therapeutic interventions.

Moreover, the integration of bioinformatics in TKI development has expedited the drug discovery and optimization process, leading to the creation of more effective and selective TKIs for cancer treatment. Although some TKIs in clinical trials have demonstrated limited specificity and efficacy, the future of TK-targeted therapeutics in GBM holds promise.
